# Bison and bovine rectoanal junctions exhibit similar cellular architecture and *Escherichia coli* O157 adherence patterns

**DOI:** 10.1186/1746-6148-9-266

**Published:** 2013-12-28

**Authors:** Indira T Kudva, Judith A Stasko

**Affiliations:** 1 Food Safety and Enteric Pathogens Research Unit, National Animal Disease Center, Agricultural Research Service, U.S. Department of Agriculture, Ames, IA 50010, USA; 2 Microscopy Services Laboratory, National Animal Disease Center, Agricultural Research Service, U.S. Department of Agriculture, Ames, IA 50010, USA

**Keywords:** O157:H7, Bovine, Bison, Tissue, Epithelia, Markers, Adherence

## Abstract

**Background:**

*Escherichia coli* O157 (*E. coli* O157) has been isolated from bison retail meat, a fact that is important given that bison meat has been implicated in an *E. coli* O157-multistate outbreak. In addition, *E. coli* O157 has also been isolated from bison feces at slaughter and on farms. Cattle are well documented as *E. coli* O157 reservoirs, and the primary site of *E. coli* O157 persistence in such reservoirs is the rectoanal junction (RAJ), located at the distal end of the bovine gastrointestinal tract. Since bison and cattle share many genetic similarities manifested as common lineage, susceptibility to infection and the nature of immune responses to infectious agents, we decided to evaluate whether the RAJ of these animals were comparable both in terms of cellular architecture and as sites for adherence of *E. coli* O157. Specifically, we compared the histo-morphologies of the RAJ and evaluated the *E. coli* O157 adherence characteristics to the RAJ squamous epithelial (RSE) cells, from these two species.

**Results:**

We found that the RAJ of both bison and cattle demonstrated similar distribution of epithelial cell markers villin, vimentin, cytokeratin, E-cadherin and N-cadherin. Interestingly, N-cadherin predominated in the stratified squamous epithelium reflecting its proliferative nature. *E. coli* O157 strains 86–24 Sm^R^ and EDL 933 adhered to RSE cells from both animals with similar diffuse and aggregative patterns, respectively.

**Conclusion:**

Our observations further support the fact that bison are likely ‘wildlife’ reservoirs for *E. coli* O157, harboring these bacteria in their gastrointestinal tract. Our results also extend the utility of the RSE-cell assay, previously developed to elucidate *E. coli* O157-cattle RAJ interactions, to studies in bison, which are warranted to determine whether these observations *in vitro* correlate with those occurring *in vivo* at the RAJ within the bison gastrointestinal tract.

## Background

Sixty million bison also referred to as buffalo, roamed North America before 1492 [1-3]. These comprised both the plains bison (*Bison bison bison*) found along the Great Plains, and the wood bison (*Bison bison athabascae*) restricted to the Northwest Territories and Alberta. However, by mid-1880, these animals became nearly extinct; their numbers reduced to 750 as a result of indiscriminate hunting for hides, meat and sport. Private herds held by ranchers and national parks enabled restoration of the bison population which were recorded at ~1 million in 2009 [1-3]. Although no longer listed as endangered, bison are still treated as a “conservation” species because of their relative low numbers, ongoing breeding and selection practices [1-3].

Bison are phylogenetically related to the European bison (*Bison bonasus*), African (*Syncerus caffer*) and Asian buffaloes (*Bubalus arnee*, *Bubalus bubalis*), yak (*Bos grunniens*, *Bos mutus*) and domesticated cattle (*Bos taurus*) [1,4]. Bison and cattle share several innate immunological features, some of which may actually help this animal combat shared diseases, most common of which are brucellosis, tuberculosis, anthrax, and malignant catarrhal fever [5-9]. While bison may acquire these infections in the wild, increased exposure has been associated with co-mingling domesticated ruminants [8-10]. Additionally, a renewed interest in the low cholesterol and high protein bison meat has resulted in these animals being actively farmed, thereby enabling transmission of disease agents among bison and other livestock [7,11]. Bison and cattle appear to share several gastrointestinal microflora, with the predominating gram-negative bacteria in fecal samples being *Escherichia coli* (*E. coli*) [12]. Studies evaluating the fecal *E. coli* serotypes indicate that while *E. coli* O157:H7 (*E. coli* O157) may not be consistently isolated from the gastrointestinal tracts of wild bison, it is prevalent in 17-83% of farmed bison much like its recovery from farmed Asian water buffaloes [12-14].

*E. coli* O157 are important foodborne, human pathogens that have been implicated in several outbreaks; an estimated 63,153 illnesses, 2,138 hospitalizations and 20 deaths occur annually in the United States [15-17]. Human disease ranges from self-limiting watery diarrhea to debilitating bloody diarrhea that can advance into often-fatal secondary sequelae in susceptible patients [18-20]. The annual cost of these human Shiga Toxin-producing *E. coli* (STEC) infections range anywhere from $26 to $211,084, depending on the severity of the disease caused [15,17,21-23]. Cattle are the primary reservoirs for *E. coli* O157 and hence, food products derived from these ruminants contaminated with *E. coli* O157-containing manure are the major sources of infection [18-20], resulting in large scale recalls of contaminated meat and produce. These recalls result in losses of up to millions of dollars annually for the meat industry [21,22]. Adding to the complexity of this situation is cross-contamination of food from sources other than cattle, many of which remain unidentified, unregulated or under voluntary federal inspection [3,11,14,20,24]. Bison is one such source; *E. coli* O157 has been isolated from bison retail meat, with variable levels of contamination [3,25,26]. Recently, *E. coli* O157-contaminated ground bison was implicated in a multi-state outbreak, resulting in the recall of 66,000 lbs of this meat [3,24,27]. Reinstein *et al.,* reported a 42.1% *E. coli* O157 prevalence in bison feces at slaughter and the high recovery in the pasture-fed bison was correlated with similar prevalence in cattle fed forage diets [28]. Given the recovery of *E. coli* O157 from bison (animal and meat) and its similarity to cattle, several studies are underway to determine *E. coli* O157 colonization patterns in this animal [25,26,28,29]. The rectoanal junction (RAJ) is the primary site of *E. coli* O157 persistence in cattle gastrointestinal tracts, but a similar observation has not been conclusively made with bison [28,30-32]. Hence, in this study, we (i) examined the cellular architecture via comparative histo-morphological studies of bison and bovine rectoanal junctions, (ii) determined whether *E. coli* O157 could adhere to squamous epithelial (RSE) cells derived from bison rectoanal junctions, and (iii) compared the patterns of *E. coli* O157 adherence to RSE cells from both bison and bovine rectoanal junctions. Our results indicate that bison are likely reservoirs of this human pathogen; it also extends the utility of the RSE cell adherence assay for confirmatory studies of the same.

## Methods

### Animals and bacteria

Eight cattle and five bison, included in unrelated experiments at the National Animal Disease Center (NADC), Ames, IA, under the approval of the NADC-Animal Care and Use Committee, were sampled in this study [32-34]. Six of the eight cattle had been experimentally inoculated with *E. coli* O157 [32-34]; a streptomycin-resistant derivative of the clinical *E. coli* O157 strain 86–24 (86–24 Sm^R^; NADC # 5570). The remaining two cattle were part of the ‘blood donor’ group that is routinely maintained at the NADC, and not exposed to *E. coli* O157. The cattle were of various breeds, ranged in age from 4 months to 8 years, and were fed according to their age, a post-weaning diet (two-thirds grain and one-third hay), or the NADC maintenance diet (corn silage, grass hay, 520 pellets (Purina Mills, St. Louis, MO), protein supplements). The five bison were approximately 2 years in age and fed prairie hay and 521 pellets (Purina Mill, St. Louis, MO), and never inoculated with *E. coli* O157. All animals had ad-libitum access to water. Rectoanal junctions (RAJ) tissue samples were recovered from all these animals at necropsy. The *E. coli* O157 strains 86–24 Sm^R^ and EDL 933 [32-34] were used in the RSE cell-adherence assays.

### Tissue sampling and histology

Each RAJ tissue sample, dissected out of both bison and bovine gastrointestinal tracts, was divided into three parts to process using different techniques. One part of the RAJ was opened and its mucosal surface placed against a piece of liver from the same animal to support and maintain the structural integrity of this surface. The tissue assembled in this manner was then placed in the OCT solution (Optimal Cutting Temperature solution Tissue-Tek, Sakura Finetek, Torrance, CA) and flash-frozen in isopentane on dry ice-ethanol bath before storing at -80°C [33]. The frozen tissue was sectioned in the laboratory using the Leica CM 1900 cryostat (Leica Microsystems, Buffalo Grove, IL), and the sections collected on Probe On + slides (Thermo Fisher Scientific, Pittsburgh, PA) were air-dried and fixed in 95% ethanol before staining [33,35]. The second part of the RAJ was fixed in 10% formalin 24–48 h, embedded in paraffin and sections prepared and processed on Probe On + slides as described before [32]. The third piece was rinsed with sterile phosphate buffered saline (PBS) and transported to the laboratory in Dulbecco’s Modified Eagle Medium -No Glucose (DMEM-NG; Invitrogen, Carlsbad, CA) supplemented with 2.5% fetal bovine serum (Thermo Scientific HyClone, Logan, UT), 100 μg/ml streptomycin- 100 U/ml penicillin (Pen-Strep; Invitrogen) and 50 μg/ml gentamicin (Invitrogen), to harvest the RAJ squamous epithelial (RSE) cells as described previously [33].

### Staining

(i) Immunofluorescent staining

Ethanol-fixed slides were processed for immunofluorescent staining using a previously described protocol [33]. Briefly, the slides were washed in PBS at room temperature and blocked with 5% normal goat serum in PBS (37°C for 30 min) prior to incubation with selected primary antibody diluted in PBS (37°C for 1 h). Subsequently, slides were washed with PBS at room temperature and incubated with the corresponding secondary antibody diluted in 5% normal goat serum in PBS (37°C for 1 h). Then the slides were washed, air dried in the dark and coverslipped with Prolong Gold antifade reagent containing the DNA stain 4′, 6′- diamidino-2-phenylindole (DAPI; Invitrogen, Carlsbad, CA). When required, slides were permeabilized with 0.1% Triton X-100 prior to staining with Phalloidin as recommended by manufacturer (Life Technologies, Grand Island, NY). Primary and the corresponding secondary antibodies used for immunofluorescent staining are shown in Table 1. All slides were analyzed using the Nikon Eclipse E800 fluorescence microscope (Nikon Instruments Inc., Elgin, IL) equipped with fluorescence illumination and digital imaging. Digital images were obtained using a Digital sight DS-Ri1camera (Nikon) and acquired using the NIS-Elements imaging software (Nikon). Controls used to verify specificity of antibody interactions included slides with no bacteria or staining with unrelated fluorescence-tagged antibodies [33].

(ii) Immunoperoxidase staining

Indirect immunoperoxidase (horseradish peroxidase) staining was done at room temperature, on formalin-fixed paraffin embedded (FFPE) tissue section slides, after de-paraffinization using standard protocols [36]. The slides were washed with tris-buffered saline (TBS), and processed as described previously [33]. Briefly, slides were blocked with the universal block (KPL, Gaithersburg, MD) and 5% normal rabbit serum (NRS) in TBS, and subsequently incubated (2 hr) with goat anti-O157 (diluted in NRS; KPL) primary antibody. After three washes with TBS, the slides were incubated with biotinylated rabbit anti-goat (diluted in NRS) secondary antibody (30 min; Vector Laboratories, Burlingame, CA) which was traced with the avidin-tracer (horseradish peroxidase)-complex solution (BioStain Super ABC Kit, Biomeda Corporation, Foster City, CA.), an enhancer solution prepared with 0.25% Brij (Thermo Fisher Scientific, Pittsburgh, PA.) in TBS, and the diaminobenzidine hydrochloride (DAB) - peroxide solution. Next, the slides were counterstained with hematoxylin (Gills No. 2; Sigma-Aldrich, St. Louis, MO), washed, dehydrated in 95% and 100% ethanol before coverslipping in a 1:1 solution of permount and xylene (Molecular Devices, Inc., Sunnyvale, CA).

**Table 1:** Antibodies used for immunofluorescence staining

**Primary/target**	**Source**	**Secondary**	**Source**
Mouse anti- (PAN) cytokeratins/Eukaryotic cytoskeletal protein	AbD Serotec, Raleigh, NC	Alexa Fluor 594 (red) labeled goat anti-mouse IgG (H + L; F (ab’)_2_ fragment)	Life Technologies, Grand Island, NY
Mouse anti- villin/Eukaryotic brush border protein	Chemicon, EMD Millipore, Billerica, MA	Alexa Fluor 488 (green) labeled goat anti-mouse IgG (H + L; F (ab’)_2_ fragment)	Life Technologies, Grand Island, NY
Mouse anti-vimentin/Eukaryotic mesenchymal cell protein	Abcam, Cambridge, MA	Alexa Fluor 488 (green) labeled goat anti-mouse IgG (H + L; F (ab’)_2_ fragment)	Life Technologies, Grand Island, NY
Rabbit anti- N-cadherin/Eukaryotic transmembrane glycoprotein	Abcam, Cambridge, MA	Alexa Fluor 594 (red) labeled goat anti-rabbit IgG (H + L; F (ab’)_2_ fragment)	Life Technologies, Grand Island, NY
Rat anti- E-cadherin/Eukaryotic transmembrane glycoprotein	Novus Biologicals, Littleton, CO	Alexa Fluor 488 (green) labeled goat anti-rat IgG (H + L; F (ab’)_2_ fragment)	Life Technologies, Grand Island, NY
Alexa Fluor 594 (red) labeled phallodin/Eukaryotic microfilament protein actin	Life Technologies, Grand Island, NY	-	-
-	-	FITC (green) labeled goat anti-O157/targets surface antigen of *E. coli* O157 bacteria	KPL, Gaithersburg, MD

### RSE cell adherence assay

Adherence of *E. coli* O157 to bovine RSE cells was previously demonstrated and developed into an adherence assay in our laboratory [33,34]. In the present study, the assay was used to compare interactions of *E. coli* O157 with RSE cells obtained from bison versus bovine RAJ. Each of the RSE adherence assays was conducted in eight technical and two biological replicates as described previously [33,34]. Briefly, RSE cells were washed and resuspended in DMEM-NG (Invitrogen) with 2.5% D + Mannose to a final concentration of 10^5^ cells/ml. Bacterial pellets from overnight cultures in DMEM-Low Glucose (OD_600_ ~0.5 – 0.7) were washed and mixed with RSE cell suspensions to a final bacteria: cell ratio of 10:1 and incubated at 37°C, 110 rpm, for 4 h. Subsequently, the mixture was pelleted, washed, reconstituted in 100 μl distilled water and eight-2 μl drops of this placed on Polysine (Thermo Scientific Pierce, Logan, UT) slides. Quenched, dried, and 95% ethanol-fixed slides were then stained with 1% toluidine blue, or fluorescence-tagged antibodies that target *E. coli* O157 and the RSE cell cytokeratins as described previously (Table 1; 33). *E. coli* O157 adherence patterns on RSE cells were recorded as either diffuse or aggregative (clumps) for all interactions that involved direct association with the cells, as described previously [33,34]. Bacterial adherence was quantitated and the average percents with standard error of means between trials was calculated using the GraphPad Prism5 software, as before [33,34]. If more than 50% of RSE cells had > 10 bacteria attached, the adherence was recorded as strongly positive. For > 50% RSE cells with 1–10 adherent bacteria, the adherence was recorded as moderately positive. For less than 50% RSE cells with 1–5 adherent bacteria, the result was recorded as nonadherent.

### Transmission Electron Microscopy (TEM)

(i) Tissue preparation

Portions of FFPE tissues were processed using a standard protocol [37,38]. After removal of paraffin, the samples were put through changes of xylene and immersed in graded alcohol solutions before being fixed with 2.5% glutaraldehyde for 1–2 h, and transferred to PBS. The samples were then post-fixed in 1% osmium tetroxide, processed through another series of graded alcohols, embedded in Eponate 12™ resin (Ted Pella, Redding, CA) and polymerized at 60°C, overnight [37,38].

(ii) RSE cells preparation

An ‘agar-block’ technique was devised that would enable capturing the RSE cells and processing them like tissue samples. The RSE cells (bison/bovine), with and without adhering *E. coli* O157, were fixed with 2.5% glutaraldehyde in 0.1 M cacodylate buffer, overnight. Next, the suspension was centrifuged; the resulting pellet was placed into molten 2% agar, and allowed to set in block-molds. The agar-blocks were then post-fixed in 1% osmium tetroxide, processed and embedded in the Eponate 12™ resin as described above [37,38].

(iii) Negative staining

Negative staining [39] of RSE cells- *E. coli* O157 suspensions was done as an initial screen to verify eukaryotic cell structure and presence of bacteria before proceeding with the agar-block preparation. Briefly, equal volumes of sample and 2% phosphotungstic acid at pH 7.0 were mixed and placed on formvar coated copper grids for 3 min. The excess liquid was wicked, grids air-dried and visualized on the FEI Tecnai™ G^2^ Biotwin electron microscope.

(iv) Immunogold staining

Thin sections of the Eponate 12™ resin blocks embedded with tissue or RSE cells with *E. coli* O157 were cut 70 nm thick on the Leica UC7 ultratome (Leica). The sections were mounted on nickel grids and processed for colloidal gold staining [38] as follows. The grids were incubated in 4% sodium metaperiodate solution (Sigma) to expose reactive antigens on bacterial surface [40]. Following multiple washes in water, the grids were incubated in 0.05 M glycine (to inactivate any background reactive aldehyde groups) and blocked with 5% Aurion bovine serum albumin in TBS, before floating them in goat anti-O157 (KPL) primary antibody diluted in TBS with 0.05% Tween20 (TBS-T). After further washes with TBS-T, the slides were incubated with Aurion 10 nm gold labeled-rabbit anti-goat (diluted in TBS-T) secondary antibody. The grids were subsequently washed and counter-stained with uranyl acetate and Reynolds lead citrate using standard protocols [39,41,42], before viewing on the electron microscope. All reagents used for TEM were obtained from Electron Microscopy Services (EMS), Hatfield, PA.

## Results and discussion

**Figure 1:**
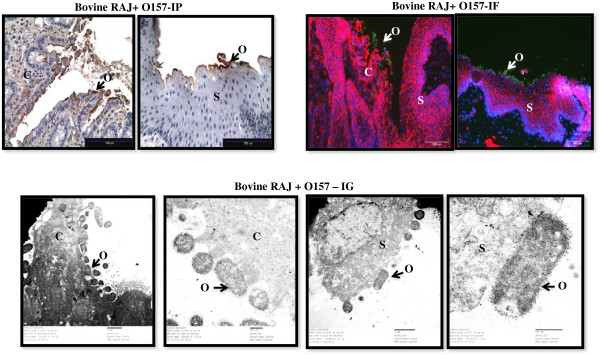
***E. coli *O157 adherence to squamous and columnar epithelium at the RAJ in experimentally inoculated *E. coli *O157-positive cattle.** Immunoperoxidase (IP), immunofluorescence (IF) and immunogold (IG) labeled RAJ tissue sections are shown. In the IP stained sections (40x magnification), epithelial cells are blue and *E. coli* O157, brown. In the IF stained sections (20x magnification), epithelial cells are orange-red with blue nuclei, and *E. coli* O157 are green. IG micrographs are shown at 6800x, 23000x, 9300x, 49000x magnifications, respectively, with *E. coli* O157 encased in 10 nm colloidal gold label. C, columnar epithelium; S, stratified squamous epithelium; O, *E. coli* O157. Arrows indicate examples of adhering *E. coli* O157.

The RAJ sections from both cattle exposed to *E. coli* O157 and those that were never exposed to *E. coli* O157 demonstrated the same histo-morphology as previously described [30,33,43] and observed by analyzing tissue sections by light, fluorescence and transmission electron microscopy. Regardless of their age and breed, the RAJ was characterized by an abrupt replacement of columnar epithelial cells by stratified squamous epithelial cells (Figure 1). The site at which this histological change occurred was either characterized by deep grooves in some animals or had planar presentation in others. Irrespective of this, *E. coli* O157 when present interacted with the cells as described previously [33,34]. *E. coli* O157 formed microcolonies on the columnar epithelial cells, about 3–5 cm from the junction, and could often be associated with local microvilli effacement as well as pedestal formations extending from the columnar epithelial cell at the site of bacterial adherence (Figure 1). Conversely, *E. coli* O157 adherence to the squamous epithelial cells was diffuse, and the bacteria appeared to adhere directly to the cell surface or pedestal-like surface structures (Figure 1).

**Figure 2:**
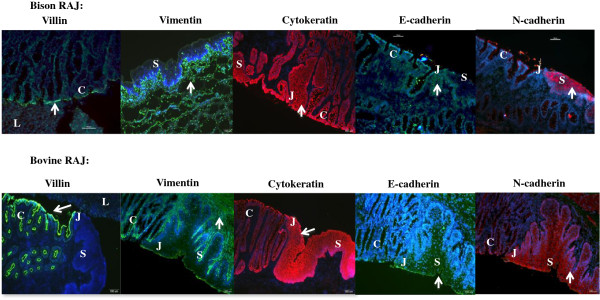
**Comparative histo-morphologies of bison versus bovine RAJ from *E. coli *O157-negative animals.** Immunofluorescence stained slides are shown at 10x magnification. Different fluorescent tags were used to detect each epithelial cell protein: Villin (green), Vimentin (green), Cytokeratin (orange-red), E-cadherein (green) and N-cadherin (red). Epithelial cell nuclei have blue fluorescence. C, columnar epithelium; S, stratified squamous epithelium; J, junction between columnar and stratified squamous epithelium; L, liver tissue section used in sample preparation. Arrows indicate representative regions of stained epithelial cell proteins.

To ascertain if bison would have similar RAJ histo-morphology as cattle, the bison and bovine RAJ was compared. As anticipated, based on the phylogenetic relationship of these animals, the cellular architecture of both RAJs were similarly characterized with the columnar epithelial cell replacement by stratified squamous epithelial cells (Figure 2). Additionally, even the various epithelial cell markers that were tested demonstrated similar distribution (Figure 2). Villin, a structural protein component of the microvilli that comprise the brush borders [44], was restricted to the columnar epithelial cells in both tissue samples, although these were more intensely detected in the bovine RAJ. This protein confers plasticity to the brush borders through its interactions with and cleavage of F-actin, the filament protein [44,45]. Vimentin is an embryonic cytoskeleton filament protein involved in the intracellular transport of proteins between the nucleus and plasma membrane [43,46]. It continues to be expressed by fibroblasts lining the submucosa, occasional M cells and carcinogenic cells in adult animals [43,46]. The protein was detected in the submucosal layers of both bison and bovine RAJ tissue sections (Figure 2). Cytokeratins are keratin containing structural proteins that extend like filaments from the surface of the nucleus to the cell membrane and contribute largely to maintaining cell-shape [47,48]. There are two types of cytokeratins, acidic and basic, which occur in pairs in an organ or tissue specific manner [47,48]. The anti-cytokeratin antibody (Table 1) preparation used in this study ensured targeting this wide range of cytokeratins that are common to all epithelial tissue [33,46-48]. Cytokeratins were found uniformly distributed in all epithelial cells comprising the RAJ of both animals (Figure 2). Cadherins comprise a superfamily of transmembrane glycoproteins involved in calcium mediated cell-cell adhesion and hence, tissue integrity [49]. Classical cadherins include epithelial-cadherin (E-cadherin), neural-cadherin (N-cadherin), placental-cadherin (P-cadherin) and vascular endothelial-cadherin (VE-cadherin). Since these cadherins appear to be broadly specific to cell-types, these proteins have also been associated with cell growth and tissue differentiation [49-52]. However, studies have reported a wider distribution of N-cadherins in non-neural tissues, of both human and bovine origin, including the mouth, breast, heart, kidney, liver, endometrium, endothelium and tumors [50,53,54]. Here we observed that both E-cadherin and N-cadherin were co-localized to the epithelial cells lining the RAJ but interestingly, N-cadherin was more abundant, especially with the squamous epithelial cells. Strong N-cadherin immunoreactivity has been associated with inflammed ileal tissues in cattle with Johne’s disease, as also with proliferative epithelium lining the healthy endometrium [50,53,54] and thus, the increased presence of N-cadherin at the RAJ may be related to the relatively rapid turn-over/growth of cells at this site. This distribution of E- and N- cadherins was similar to both bison and bovine RAJ tissue samples.

**Figure 3:**
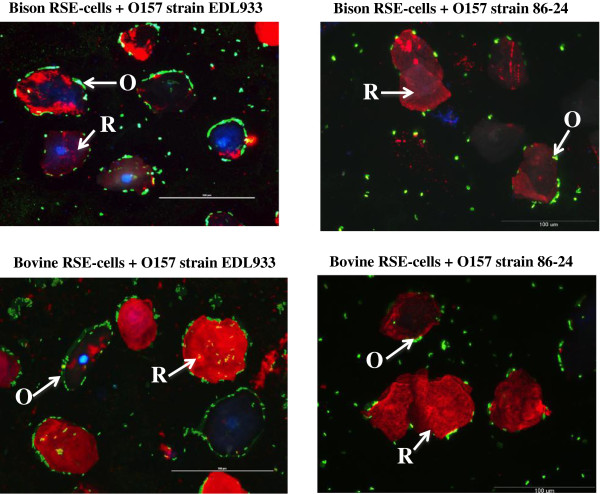
***E. coli *O157 adherence patterns on bison and bovine RSE cells in the presence of D + Mannose.** Immunofluorescence stained slides are shown at 40x magnification, with the RSE cells’ cytokeratins having orange-red, their nuclei, blue and *E. coli* O157, green fluorescence. Arrows indicate R, RSE cells and O, *E. coli* O157.

**Figure 4:**
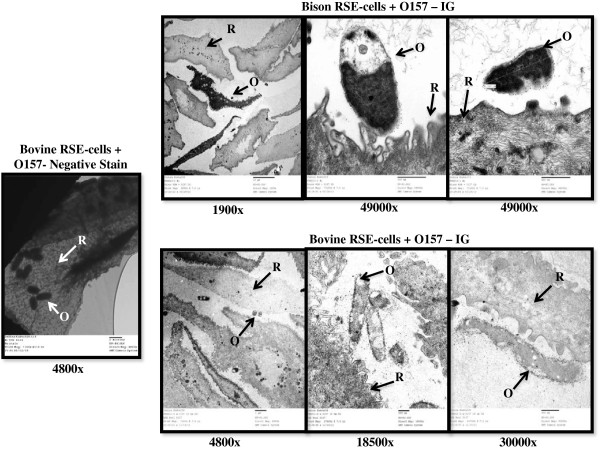
**Transmission electron micrographs confirming *E. coli *O157 adherence to bison and bovine RSE cells.** A sample of negative stained grid and other immunogold labeled grids with RSE cells and adhering *E. coli* O157 are shown; magnifications used are below each micrograph. Arrows indicate R, RSE cells and O, *E. coli* O157.

Since the bison and bovine RAJ presented several anatomical and cytological similarities, we evaluated the ability of *E. coli* O157 to adhere to bison RAJ cells. In our previous studies, we had standardized an *in vitro E. coli* O157 adherence assay using harvested bovine RAJ squamous epithelial (RSE) cells [33,34]. An in-depth analysis showed that this assay with bovine RSE cells successfully reproduced the *E. coli* O157 adherence patterns as seen *in vivo* on the RAJ of cattle infected with O157 (Figures 1, 3, 4). Hence, we extrapolated the assay to include bison RSE cells as a presumed reflection of *E. coli* O157-RAJ interactions *in vivo* in the bison. Both *E. coli* O157 strains (EDL 933 and 86–24 Sm^R^) adhered to the bison RSE cells; the binding characteristics resembled those seen with bovine RSE cells (Figure 3; Table 2). Except for the *E. coli* O157 strain 86–24 Sm^R^ strain demonstrating diffuse, moderate adherence with bison RSE cells, *E. coli* O157 strain EDL 933 bound RSE cells from both species in an aggregative, moderate pattern (Table 2). *E. coli* O157 adherence to bison RSE cells was verified and compared to bovine RSE cells using fluorescent and transmission electron microscopy (Figures 3 and 4) further confirming the true adherence of *E. coli* O157 to these RSE cells.

**Table 2:** **Quantitation of bison and bovine RSE cells with adherent *E. coli *O157 in the presence of D + mannose**

**Bacteria tested**	**Bacterial adherence pattern**	**Eukaryotic cells with adherent bacteria, in the ranges shown, for two different trials**^**1**^				**Percent Mean +/- standard error of mean, of eukaryotic cells with adherent bacteria in the ranges shown**^**5**^	
		**(MOI**^**2 **^**= 10**^**6 **^**bacteria: 10**^**5 **^**cells)**					
		**Trial I**		**Trial II**			
		**>10**	**1-10**^**(3)**^	**>10**	**1-10**	**>10**	**1-10**
Bison RSE cells + *E. coli* O157 strain EDL 933	Aggregative,	58	102	29	124	27 ± 9	**71 ± 7**
	Moderate	(160)^4^	(160)	(160)	(160)		
Bison RSE cells + *E. coli* O157 strain 86–24 Sm^R^	Diffuse,	12	138	5	140	5.5 ± 2.5	**93.5 ± 3.5**
	Moderate	(153)	(153)	(145)	(145)		
Bovine RSE cells + *E. coli* O157 strain EDL 933	Aggregative,	52	92	18	135	22 ± 11	**72 ± 13**
	Moderate	(156)	(156)	(160)	(160)		
Bovine RSE cells + *E. coli* O157 strain 86–24 Sm^R^	Diffuse,	104	53	106	28	**66.5 ± 1.5**	25.5 ± 7.5
	Strong	(160)	(160)	(155)	(155)		

^1^Each trial had one slide per bacterial group. Each slide in turn had 8 technical replicates (eight 2 spotted; 10–20 well-dispersed cells were evaluated per spot or chamber).

^2^MOI, multiplicity of infection.

^3^Number of bacteria adhering to each cell is shown as a range of >10, and 1–10. Number of cells without bacteria is not shown.

^4^Total number of cells evaluated in each trial is shown in parenthesis.

^5^Percent means for ranges used to determine “moderate or strong” adherence are in bold.

## Conclusion

Our study demonstrates that the bison RAJ shares histo-morphological characteristics with the bovine RAJ. These overlapping features may have contributed to the ability of *E. coli* O157 to adhere to bison RSE cells, thus extending this utility of the RSE cell adherence assay for incorporation in bison studies. Given that *E. coli* O157 has been isolated from bison retail meat, *E. coli* O157-contaminated bison meat has been implicated in an outbreak and that *E. coli* O157 has been isolated from these animals, this study supports that bison can serve as reservoirs for *E. coli* O157 in the wild. Experimental studies in bison are being planned to determine whether *E. coli* O157-bison RSE cell interactions seen *in vitro* occur *in vivo* as well.

## Competing interests

The authors declare that they have no competing interests.

## Author’s contributions

ITK was the project leader and designed, coordinated, conducted experiments, analyzed results, and drafted the manuscript. JAS assisted in design of experiments, data analysis, and contributed to the final draft of the manuscript. Both authors read and approved the final manuscript.
